# Synthesis of a Novel Fluorescent Ruthenium Complex with an Appended Ac_4_GlcNAc Moiety by Click Reaction

**DOI:** 10.3390/molecules23071649

**Published:** 2018-07-06

**Authors:** Qi Cheng, Yalu Cui, Nao Xiao, Jishun Lu, Chen-Jie Fang

**Affiliations:** 1 Beijing Area Major Laboratory of Peptide and Small Molecular Drugs, Engineering Research Center of Endogenous Prophylactic of Ministry of Education of China, School of Pharmaceutical Sciences, Capital Medical University, Beijing 100069, China; qicheng@ccmu.edu.cn (Q.C.); cjfang@ccmu.edu.cn (C.-J.F.); 2 Key Laboratory of Systems Biomedicine (Ministry of Education) and Collaborative Innovation Center of Systems Biomedicine, Shanghai Center for Systems Biomedicine (SCSB), Shanghai Jiao Tong University, 800 Dongchuan Road, Shanghai 200240, China; cuiyalu2011@126.com (Y.C.); lujishun@sjtu.edu.cn (J.L.)

**Keywords:** *O*-GlcNAc, click chemistry, cell imaging, fluorescent labeling, living cells

## Abstract

The *O*-linked β-*N*-acetylglucosamine (*O*-GlcNAc) modification is an abundant post-translational modification in eukaryotic cells, which plays a fundamental role in the activity of many cells and is associated with pathologies like type II diabetes, Alzheimer’s disease or some cancers. However, the precise connexion between *O*-GlcNAc-modified proteins and their function in cells is largely undefined for most cases. Confocal microscopy is a powerful and effective tool for in-cell elucidation of the function of biological molecules. Chemical labeling of non-ultraviolet or non-fluorescent carbohydrates with fluorescent tag is an essential step that makes intra-cellular microscopic inspection possible. Here we report a strategy based on the 1,3-dipolar cycloaddition, called click chemistry, between unnatural *N*-acetylglucosamine (GlcNAc) analogues Ac_4_GlcNAc (substituted with an azido group) and the corresponding fluorescent tag Ru(bpy)_2_(Phen-alkyne)Cl_2_ (**4**) to synthesize the fluorescent dye Ru(bpy)_2_(Phen-Ac_4_GlcNAc)Cl_2_ (**5**) under mild and neutral reaction conditions. Moreover, **5** showed good stability, desirable fluorescence characteristics, and exhibited rather low levels of cytotoxicity against sensitive MCF-7 cells. Additionally, we have achieved successful fluorescent imaging of **5** transported in living MCF-7 cells. Cell images displayed that proteins are potentially labelled with **5** in the cytoplasm.

## 1. Introduction

*O*-Linked β-*N*-acetylglucosamine (*O*-GlcNAc) glycosylation is an important regulation for the diverse cellular processes including transcription, cell division, and glucose homeostasis [1,2,3,4,5]. Therefore, understanding the glycosylation of proteins is critical to elucidate the functional roles of proteins within the dynamic environment of cells in physiological and diseased states [6,7,8,9]. Currently, glycosylation levels are typically monitored by immunoblotting with a general *O*-GlcNAc antibody. Due to the lack of specificity and selectivity of the antibody used, only a limited number of *O*-GlcNAc proteins can be detected [10]. With this regard, great challenge remains to develop simple but efficient methods to monitor non-ultraviolet or non-fluorescent *O*-GlcNAc glycosylation in response to cellular stimuli [11].

**Scheme 1:**
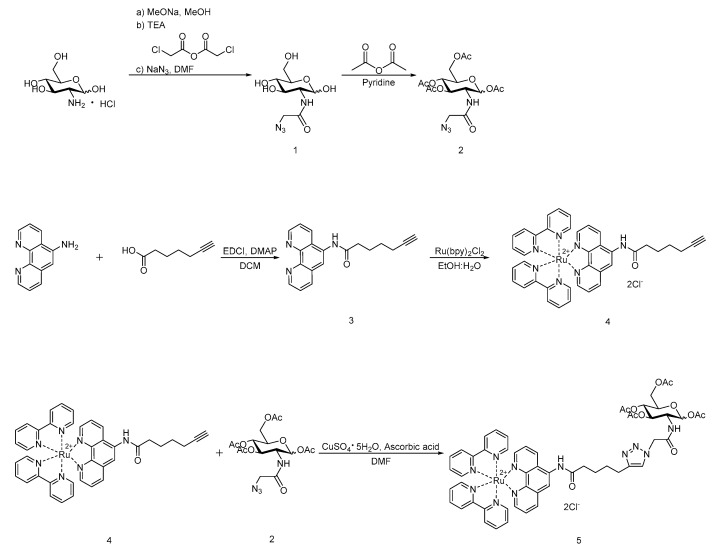
Synthesis of Ac_4_GlcNAz (**2**), Ru(bpy)_2_(Phen-alkyne)Cl_2_ (**4**) and Ru(bpy)_2_(Phen-Ac_4_GlcNAc)Cl_2_ (**5**).

Microscopic inspection is a powerful and widely used tool for high-resolution and non-invasive imaging of target molecules in their native environments [12]. With spectroscopic probes such as alkyne-functionalized rhodamine or 1,8-naphthalimide derivatives, imaging of *O*-GlcNAc proteins in their native environment is feasible [13,14,15,16]. This method definitely facilitates and advances proteomic studies by helping monitor the expression and dynamics of *O*-GlcNAc-glycosylated proteins in cells and tissues [17]. Herein, we report an alternative fluorescent dye **5** (Scheme 1) as a potential labeling reagent, which utilizes the intrinsic reactivity of the Ac_4_GlcNAc moiety at end of a ruthenium complex to specifically address the target protein by either chemical or enzymatic reactions [18,19]. This is the first example of the use of a ruthenium complex appended with the glycosylation reagent Ac_4_GlcNAc to image proteins within cells. We anticipate that this fluorescent dye will be a powerful tool for facilitating our understanding of the physiological functions and dynamic regulation of *O*-GlcNAc glycosylation within cells. The monoglucosylated complex **5** was characterized with IR, UV-vis, ^1^H-NMR, ^13^C-NMR, fluorimetry, and mass spectrometry. Further the use of complex **5** for intracellular imaging was tested with MCF-7 cells, which suggested the accumulation of **5** in the cytoplasm.

## 2. Results

The synthetic routes to the intermediates and the target ruthenium complex **5** are summarized in Scheme 1. Compound **5** was synthesized by copper-catalyzed azide-alkyne cycloaddition (CuAAC) reaction of alkyne-dye **4** and azide-based *O*-GlcNAc glycosylation metabolite Ac_4_GlcNAz (**2**). Compound **5** has trivial cytotoxicity (IC_50_ = 549 μM) against living cells under mild and neutral conditions (37 °C, 1 h). According to microscope imaging, **5** was mostly located in the cytoplasm and not the nucleus of living MCF-7 cells, which provides direct imaging and visualization of **5** transported in living cells, showing its potential as a tool for biological function elucidation of *O*-GlcNAc modified proteins.

## 3. Discussion

### 3.1. Synthesis of ***2*** and ***3***

Peracetylated *N*-azidoacetylglucosamine **2** was synthesized starting from glucosamine hydrochloride (Scheme 1). The monosaccharide was deprotonated with sodium methoxide, followed by acetylation with chloroacetic anhydride in methanol. Displacement of the halide with sodium azide in dry DMF provided the compound **1**. The acetyl protected target **2** was obtained by acetylation of **1** with acetic anhydrate in pyridine. Ligand **3** appended with an alkynyl chain was obtained with the reagents 1,10-phenanthrolin-5-amine and 6-heptynoic acid in a DCM solution of EDCI/DMAP. GlcNACl, **1**, **2**, and **3** were fully characterized by FTIR, ^1^H-NMR (Figures S1, S3 and S5), ^13^C-NMR (Figures S2, S4 and S6) and mass spectroscopy (Figures S11–S14, S16 and S17).

In the FTIR spectrum (Figure S18), a characteristic stretching band at around 2100 cm^−1^ appeared, indicating the introduction of the azido group together with the glucosyl group to form **2**, which completely disappeared after the click reaction with terminal alkyne **4**. It suggested the ruthenium complex unit was successful conjugated with the glucosyl unit.

To provide further insight into the structure of GlcNACl, **1**, **2** and **3**, ESI-MS measurements were carried out. For GlcNACl, the main peak at 277.64 (*m*/*z*) (Figure S11) is in good agreement with the theoretical molecular-ion of the sodium-cationized GlcNACl (C_8_H_14_ClNNaO_6_: 278.04), the peaks nearby main peak at 279.57, 280.17, and 280.50 are assigned to isotope forms of sodium-cationized GlcNACl. For **1**, the peaks at 243.25, and 258.75 (*m*/*z*) (Figure S12) are in a good agreement with the theoretical molecular-ions of [M-N_3_]^+^ (C_8_H_14_NNaO_6_: 243.07), and [M-N_2_]^+^ (C_8_H_14_N_2_NaO_6_: 257.07), receptivity. For **2**, the peaks at 452.82, and 883.04 (*m*/*z*) (Figure S13) are in a good agreement with the theoretical molecular-ions of [M + Na]^+^ (C_16_H_22_N_4_NaO_10_: 453.12) and [2M + Na]^+^ (C_32_H_44_N_8_NaO_20_: 883.26) without any unreacted material peaks or byproducts. For **3**, the peak at 303.81 (*m*/*z*) (Figure S14) is in a good agreement with the theoretical molecular-ions of [M + H]^+^ (C_19_H_17_N_3_O: 304.14) without any unreacted material peaks or byproducts.

Overall, FTIR, ESI-MS, and NMR results (see Supplementary Information) clearly evidenced that the **2** and **3** were successfully synthesized by acetylation or nucleophilic substitution reactions. The detailed characteristic ^1^H-NMR and ^13^C-NMR spectra of **1**, **2** and **3**, are provided in Supplementary Figures S1–S6.

### 3.2. Synthesis of Ruthenium Complexes

The ruthenium complexes of **4** and **5** (where bpy = 2,2′-bipyridine) complexes (cationic forms are shown in Scheme 1) were synthesized by efficient methods. As shown in Scheme 1, the alkynyl chain with a bidentate phenanthroline ligand **3** reacted with ruthenium precursor complex *cis*-Ru(bpy)_2_Cl_2_ in CH_2_Cl_2_:MeOH (1:1) under reflux to produce the heteroleptic complex **4**.

CuAAC is a popular strategy in biological applications such as labeling proteins and nucleic acids in situ with minimal undesired side reactions [20,21,22]. Both azide and alkyne groups are stable, and it is easy to introduce them into biomolecules or probe molecules without dramatically altering their function or metabolic processes [23,24,25,26]. Through the CuAAC method, the terminal alkyne fluorescent tag **4** is further reacted with peracetylated glucosyl moieties bearing the azido-ethyl linker **2** overnight. The catalytically active Cu^I^ was obtained with reducing Cu^II^ (CuSO_4_) by ascorbic acid in DMF solution. After all the azides were converted into the triazole-substituted adduct **5**, the solvent was removed by evaporation, and the desired **5** was obtained as the sole product in nearly quantitative yields. Both designed ruthenium complexes were achieved by only a very simple, rapid, and efficient one step reaction. The purity of prepared ruthenium complexes residue was examined by thin-layer chromatography (TLC), and the raw materials and byproducts in the residue were then removed by column chromatography on silica with MeCN:H_2_O:KNO_3_ (100:7.5:0.5) as eluent. **4** and **5** were further fully characterized with HPLC, FTIR, ^1^H-NMR, ^13^C-NMR, and mass spectroscopy.

Typically, the synthesized target ruthenium complex **5** was determined by HPLC, which showed a single peak with no impurities detected. The HPLC traces confirmed the formation of **5**, as shown in Figure S15. The pure products were later ascertained to be **5** by mass spectroscopy and NMR spectroscopy. The calculated and experimental results of the mass spectra of **4** and **5** are given in Figures S16 and S17, respectively. The calculated molar masses is 717.18 g/mol for Ru(bpy)_2_(Phen-alkyne)^2+^ and 1147.31 g/mol for Ru(bpy)_2_(Phen-Ac_4_GlcNAc)^2+^. Each ruthenium complex has an overall charge of +2, so the calculated molar masses of *m*/*z* (mass/charge) are 358.59 g/mol and 573.655 g/mol for Ru(bpy)_2_(Phen-alkyne)^2+^ and Ru(bpy)_2_(Phen-Ac_4_GlcNAc)^2+^, respectively. The measured molecular-ion peaks appear at 358.07 (Figure S16, Calcd. for C_39_H_33_N_7_ORu/2: 358.59) and 573.06 (Figure S17, Calcd. for C_55_H_55_N_11_O_11_Ru/2: 573.655) are exactly consistent with the calculated molar masses, confirming the identity and the integrity of the complexes. As expected, no other byproducts were observed in the MS profile, suggesting a clean reaction.

Furthermore, the structural information of **4** and **5** were investigated by ^1^H-NMR measurements. In the analysis of Figure S19 (upper), the proton signals of a’–e’ due to the methyne and alkynyl on **4** were clearly observed in the range of 1.63–2.76 ppm, and the signals of the aromatic part assignable to the bpy and phenanthroline segments were simultaneously observed. The click reaction was also further verified by ^1^H-NMR measurements. The ^1^H-NMR spectrum of **5** is shown in Figure S19 (lower), the new proton signals due to the glucosyl and methyl group were observed in the range of 4.02–6.13 ppm and 1.81–2.27 ppm, respectively. Additionally, the signals of the methylene protons of a’ and b’ on the alkyl chain of **4** were fully shifted from 2.84 ppm to 2.69 ppm after the click reaction. The observed protons are consistent with the presence of protons on ruthenium complexes structures by integration for each signal. These results indicated the formation of the Ru(II) complex **4** and **5**.

Data from ESI-MS, HPLC, ^1^H-NMR, and ^13^C-NMR indicated that the **4** could be quantitatively conjugated with **2** to afford **5** via click reactions with no byproducts, implying its potential in fluorescent labeling of proteins modified with *O*-GlcNAc. Detailed characteristic ^1^H-NMR and ^13^C-NMR spectra of **4** and **5**, are presented in Supplementary Figures S7–S10.

### 3.3. UV-Vis and Fluorescence Properties Studies of ***4*** and ***5***

**Figure 1:**
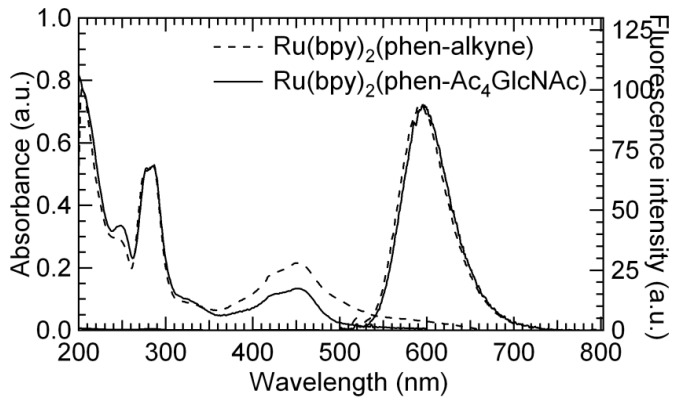
UV-vis absorption and fluorescence spectra of **4** and **5** in MeOH. *λ*_ex_ = 450 nm.

To explore the optical properties of **4** and **5**, the electronic absorption and emission spectra were recorded, as shown in Figure 1. Compounds **4** and **5** displayed maxima excitation and emission wavelength at 450 nm and 590 nm, respectively, indicating that the inherently favorable spectroscopic and photophysical properties of the parent complex are not substantially altered by the ligand substitution. The absorbance peak appeared around 450 nm is caused by the metal-to-ligand charge transfer (MLCT) effect in the ruthenium complex core, which resulted in the slightly red color of the aqueous solution. Both complexes exhibit a single emission band. The observed emission spectra of the complexes are very close to that of the ruthenium complex previously reported by Yuan’s group [27].

As shown in Figure S20 and Table S1, both ruthenium complexes **4** and **5** underwent a signal-exponential decay with almost the same lifetime of around 190 ns. The good fluorescence properties implied the potential to imaging of **2** transported within living cells.

### 3.4. Cytotoxicity of ***5*** against Living Cells

**Figure 2:**
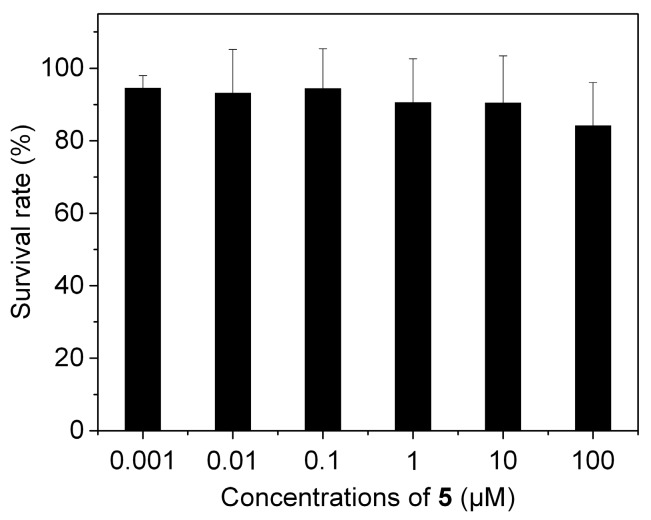
Cytotoxicity of **5** against MCF-7 cells.

MCF-7 cells were adopted since they were used for studying the labeling *O*-GlcNAc-modified proteins in the past [28,29]. In terms of fluorescent imaging in living cells, the fluorescent tags should be friendly to living cells. To ascertain whether the imaging reagent might alter the cells’ biological function, the survival rates of cells administered with **5** was determined by a standard 3-(4,5-dimethylthiazolyl-2)-2,5-diphenyltetrazolium bromide (MTT) analysis (Figure 2). Cells incubated with medium only were used as control. At concentrations ranging in 0.001 to 100 μM, the survival rates of the cells was higher than 80%, suggesting **5** has hardly any negative effect on the proliferation of living cells. The IC_50_ value of **5** is 549 μM (Figure S21), which also suggested a trivial cytotoxicity of **5** against MCF-7 cells.

### 3.5. Imaging of ***5*** against Living MCF-7 Cells

**Figure 3:**
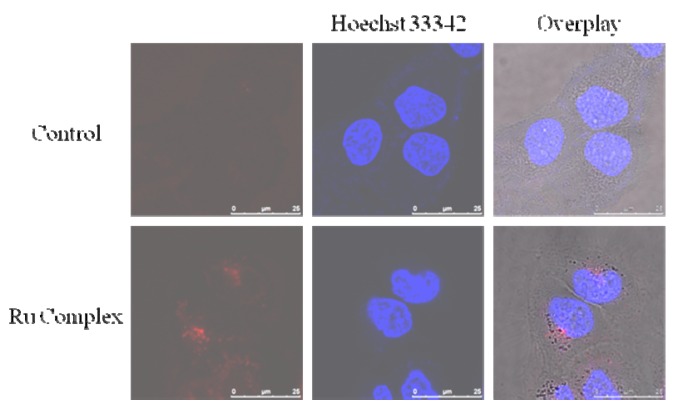
Confocal microscopy images of MCF-7 cells subjected to ligation with **5**, followed by staining with Hoechst 33342 (blue, nucleus-specific dye). The overlay indicates the colocalization of ruthenium complex **5** and the Hoechst 33342.

Imaging of **5** transported in living MCF-7 cells, which were cultured with **5** in PBS for 24 h at 37 °C and 5% CO_2_, followed by stained with Hoechst 33342 in PBS (10 mg/mL) for 10 min was performed. To track the sub-cellular location of the target molecule **5** transported within living cells, these treated MCF-7 cells were imaged under confocal microscopy at 20× by two-channel scanning. As shown in Figure 3, compound **5**-tagged proteins appeared as red fluorescent spots that were predominantly located not in the nucleus but inside the cytoplasm of MCF-7 cells (left panel).

Hoechst 33342 (nucleus specific dye, middle panel) dyed proteins appreared as blue fluorescent spots that were dominantly located in the nucleus of MCF-7 cells. Overlapping of the fluorescent image profiles (right panel) deduced from co-localization with 5 and Hoechst 33342 imaging further demonstrated that label 5 was basically located in cytoplasm of the MCF-7 cell line, indicating its potential utility in sub-cellular localizations of the *O*-GlcNAc-glycosylation within living cells. Apparently, the imaging profiles demonstrated that the 5 could be desirably used as a fluorescent tags, which enables further fluorescence microscopy studies of *O*-GlcNAc-glycosylation modified proteins such as visualization of imaging, locating or tracking of target molecules within living cells

## 4. Materials and Methods

### 4.1. Materials

All chemicals and reagents were of analytical grade or of the highest purity available and were used without further purification. Ultrapure water was used throughout the experiments. D(+)-Glucosamine hydrochloride, sodium methylate (MeONa), chloroacetic anhydride, *N*,*N*-dimethylformamide (DMF), methanol (MeOH), triethylamine, pyridine, dichloromethane (DCM), ethanol (EtOH), 1,10-phenanthrolin-5-amine (Phen-NH_2_), 4-dimethylaminopyridine (DMAP), 6-heptynoic acid, the complex (*cis*-bis-(2,2′-bipyridine)dichlororuthenium dihydrate, (*cis*-Ru(bpy)_2_Cl_2_·2H_2_O), copper sulfate pentahydrate (CuSO_4_·5H_2_O), ascorbic acid, potassium nitrate (KNO_3_), and 1-(3-dimethylaminopropyl)-3-ethylcarbodiimide hydrochloride (EDC·HCl, or EDC) were purchased from Aladdin Reagent (Shanghai, China), fetal bovine serum from ExCell Biology, Inc. (Shanghai, China), and 1% antibiotics from Solarbio (Beijing, China). TLC analyses were performed on silica gel plates and column chromatography was conducted over silica gel (mesh 200–300), both of which were obtained from the Qingdao Haiyang Chemicals (Qingdao, China). A semi-preparative column (Dikma Tech. Inc., Beijing, China, Luster C18, 10 mm, 21.2 × 250 mm) was used to purify ruthenium complexes. The purities of ruthenium complex **5** (>98%) and the intermediates (>95%) were measured by TLC analysis (Qingdao silica gel plates of GF254, 0.25 mm layer thickness) and HPLC analysis (XTerra^®^ MS C18, 5 μm, 2.1 × 150 mm, Waters, Milford, MA, USA). UV-vis absorption spectra were recorded on a UV-2550 spectrophotometer (Shimadzu, Kyoto, Japan). Photoluminescence was recorded with a F-2500 fluorescence spectrometer (Hitachi, Tokyo, Japan) with the excitation and emission slit widths at 5.0 nm and the excitation wavelength at 450 nm. Fluorescence lifetimes were detected by a FLS980 fluorescence spectrometer from Edinburgh Instruments. The monitored wavelength was 590 nm. The fitting parameters (decay times and pre-exponential factors) were decided by minimizing the reduced chi square χ^2^. IR spectra were recorded using a FT-IR spectrometer (Thermo Nicolet IS5, Thermo Fisher Scientific, Waltham, MA, USA). Approximately 1 mg of sample was measured using the KBr pellet method at room temperature. ^1^H-NMR (300 MHz) and ^13^C-NMR (75 MHz) spectra were recorded on an INOVA-300 MHz spectrometer (Varian, Palo Alto, CA, USA) with MeOD as the solvent and tetramethylsilane as internal standard. ESI/MS spectra were recorded on ZQ 2000 (Waters) and solariX FT-ICR mass spectrometer (Bruker Daltonics, Breman, Germany) with an ESI/MALDI dual ion source and 9.4 T superconductive magnet.

### 4.2. Synthesis of 2-Azidoacetamido-2-deoxy-β-d-glucopyranose (GlcNAz, ***1***)

Compound **1** was synthesized through a modification of a previously reported procedure [30]. Glucosamine hydrochloride (800 mg, 3.71 mmol) was dissolved in MeOH (10 mL), to which an equivalent amount of sodium methoxide (200 mg, 3.71 mmol) had been added. The reaction mixture was stirred at room temperature for 4 h, and then triethylamine (0.57 mL, 4.11 mmol) and chloroacetic anhydride (1.500 g, 8.77 mmol) were added. The reaction mixture was stirred for another 6 h. Removal of the solvent provided the corresponding chloro intermediate, which was partially purified by silica gel chromatography eluting with a gradient of CH_2_Cl_2_:MeOH (6:1 to 4:1). The resulting oil was dissolved in DMF (20 mL), NaN_3_ (2.0 g, 33.33 mmol) and 15-crown-5 (100 μL) were added and the reaction mixture was heated to 80 °C for 2 h. After cooling to room temperature, the solvent was removed under reduced pressure and the residue was purified by silica gel column chromatography (CH_2_Cl_2_:MeOH 5:1) to give the title compound as a light yellow oil (600 mg, 2.29 mmol, 62% over two steps).

### 4.3. Synthesis of 1,3,4,6-Tetra-O-acetyl-2-N-azidoacetyl-2-deoxy-β-d-glucopyranose (Ac_4_GlcNAz, Mixture of Anomers, ***2***)

Compound **2** was synthesized through a modification of a procedure reported in the literature [31]. Compound **1** (524 mg, 2 mmol) was dissolved in pyridine (16 mL) and Ac_2_O (8 mL). After stirring for 12 h at room temperature, the solvent was removed under reduced pressure and the residue was dissolved in CH_2_Cl_2_ (50 mL). The organic solution was washed with H_2_O (3 × 100 mL), dried over anhydrous Na_2_SO_4_, concentrated, and purified by silica gel chromatography eluting with CH_2_Cl_2_:MeOH (5:1) to afford **2** as a lear thick oil (632 mg, 1.47 mmol, 73.5%).

### 4.4. Synthesis of N-(1,10-phenanthrolin-5-yl)hept-6-ynamide (Phen-alkyne, ***3***)

Compound **3** was synthesized through a modification of a procedure reported in the literature [32]. 5-Amino-1,10-phenanthroline (488 mg, 2.50 mmol, 1 eq.) was dissolved in DCM (20 mL) at room temperature. 6-Heptynoic acid (315 mg, 2.50 mmol, 1 eq.) was added, followed by 1-ethyl-3-(3-dimethylaminopropyl)carbodiimide hydrochloride (EDCI, 959 mg, 5.00 mmol, 2.0 eq.) and finally 4-dimethylaminopyridine (DMAP, 305 mg, 2.50 mmol, 1 eq.). The mixture was allowed to stir for 24 h. The solvent was removed under reduced pressure before H_2_O (50 mL) was added causing precipitation of a white solid which was isolated by filtration. The resulting solid was redispersed in MeCN (10 mL) and again filtered before being collected by filtration under vacuum and dried under high vacuum to give a beige/white solid (637 mg, 2.10 mmol, 84.0%).

### 4.5. Synthesis of Ru(bpy)_2_(Phen-alkyne)Cl_2_ (***4***)

Ligand **3** (152 mg, 0.5 mmol, 1 eq.) and *cis*-Ru(bpy)_2_Cl_2_·2H_2_O (260 mg, 0.5 mmol, 1 eq.) were suspended in EtOH:H_2_O (8:2), and the suspension was degassed by bubbling with argon for 15 min and heated at 160 °C for 8 h. After cooling to room temperature, the reaction mixture was filtered through a funnel, the solvent was removed under reduced pressure, and the residue was purified by silica gel column chromatography eluting with MeCN:H_2_O:KNO_3_ (100:7.5:0.5). The fractions containing compound **4** was collected, and the solvent was evaporated. The resulting solid was dissolved in CH_3_CN to remove the excess KNO_3_ by filtration to give compound **4** as a red solid (262 mg, 0.37 mmol, 73.18%).

### 4.6. Synthesis of Ru(bpy)_2_(Phen-Ac_4_GlcNAc)Cl_2_ (***5***)

DMF (20 mL) was added into a round bottomed flask containing **2** (52.0 mg, 0.12 mmol), **4** (72.0 mg, 0.1 mmol), copper (II) sulfate pentahydrate (7.5 mg, 0.03 mmol) and ascorbic acid (17.6 mg, 0.1 mmol). The mixture was stirred at room temperature overnight. The solvent was removed under reduced pressure, and the residue was purified by silica gel chromatography eluting with a 100:7.5:0.5 MeCN:H_2_O:KNO_3_ ratio. The fractions containing **5** were collected, and the solvent was evaporated. The resulting solid was dissolved in CH_3_CN to remove the excess KNO_3_ by filtration to give compound **5** as a red solid (103.25 mg, 0.09 mmol, 90.00%).

### 4.7. Evaluation of the Cell Viability and Metabolic Activity

The survival rates of cells administered with free **5** was determined by a standard 3-(4,5-dimethylthiazolyl-2)-2,5-diphenyltetrazolium bromide (MTT) analysis. In brief, the MCF-7 cells were seeded in a 96-well microplate for 18 h and cultured with **5** at various concentrations for 36 h, supplemented with 10% (*v*/*v*) fetal bovine serum, 1% antibiotics, and cultured at 37 °C in an atmosphere of 5% CO_2_. Meanwhile, the cells treated with medium only were used as control. Then, the 20 μL of 0.5 mg/mL MTT solution was added into each well for 4 h. The incubation was suspended by removing the medium and adding 100 μL of DMSO. After a 15-min gentle vortex, the light absorption value of each well was recorded at 490 nm on an Infinite M 200 Pro Multilabel Plate Reader (Tecan, Mannedorf, Switzerland).

### 4.8. Imaging Cellular Uptake of ***5***

Cellular uptake and distribution of **5** was observed by TCS SP5 confocal microscopy (Leica Microsystems, Wetzlar, Germany). MCF-7 cells were incubated on cell culture dishes at a density of 1 × 10^5^ cells per dish for 16 h with **5** (100 μmol/mL, 2 mL), supplemented with 10% (*v*/*v*) fetal bovine serum, 1% antibiotics, and cultured at 37 °C in an atmosphere of 5% CO_2_. After removing drugs and washing the dishes twice with PBS, the cell nuclei were then stained with Hoechst 33342 in PBS (10 mg/mL) for 10 min. The following wavelengths were used: excitation at 346 nm and detection through a 460 nm filter for Hoechst 33342, and excitation at 450 nm and detection through a 593 nm filter for **5**.

## 5. Conclusions

We demonstrate here a robust and general method to synthesize a novel water-soluble ruthenium dye **5** bearing an Ac_4_GlcNAc moiety by a click reaction, which could be used in living cell imaging. Our results indicated that the sugar-derived **5** displayed similar fluorescence properties to its precursor **4**, and could be delivered into the inside of cells through the fat-soluble cell membrane. The IC_50_ of **5** against living MCF-7 cells is 549 μM. By microscope imaging, compound **5** was mostly located in the cytoplasm and not the nucleus of living MCF-7 cells, which provides direct insight into the imaging and visualization of **5** transported in living cells, showing its potential for biological function elucidation of *O*-GlcNAc-modified proteins. According to the results, the dye-Ac_4_GlcNAc reagent **5** might has potential applications as a metabolic labeling reagent. The bioconjugation reactions between **5** and relevant proteins are underlined within living cells.

## Supplementary Materials

The following are available online. Figure S1: ^1^H-NMR spectrum of **1** in MeOD, Figure S2: ^13^C-NMR spectrum of **1** in MeOD, Figure S3: ^1^H-NMR spectrum of **2** in CDCl_3_, Figure S4: ^13^C-NMR spectrum of **2** in CDCl_3_, Figure S5: ^1^H-NMR spectrum of **3** in CDCl_3_, Figure S6: ^13^C-NMR spectrum of **3** in CDCl_3_, Figure S7: ^1^H-NMR spectrum of **4** in MeOD, Figure S8: ^13^C-NMR spectrum of **4** in MeOD, Figure S9: ^1^H-NMR spectrum of **5** in MeOD, Figure S10: ^13^C-NMR spectrum of **5** in MeOD, Figure S11: ESI-MS spectrum of GlcNACl in CH_3_OH, Figure S12: ESI-MS spectrum of **1** in CH_3_OH, Figure S13: ESI-MS spectrum of **2** in CH_3_OH, Figure S14: ESI-MS spectrum of **3** in CH_3_OH, Figure S15: HPLC trace of **5** determined in CH_3_CN/H_2_O/HAc (60:36:4). *λ* = 450 nm, Figure S16: ESI-MS spectrum of **4** in CH_3_OH. Figure S17: ESI-MS spectrum of **5** in CH_3_OH. Figure S18: IR spectra of glucosamine hydrochloride, **1**, **2** and **5**. Figure S19: ^1^H-NMR spectra of **4** and **5** in MeOD. Figure S20: The fluorescent decay curves (collected at 590 nm) of **4** and **5** in MeOH solution. Figure S21: In vitro cell viability after incubation of MCF-7 cells with **5**. Table S1: Photophysical properties of **4** and **5** were recorded in MeOH solution at room temperature.
