# Supplementary material for: Synthesis of a Novel Fluorescent Ruthenium Complex with an Appended Ac4GlcNAc Moiety by Click Reaction
Source: Molecules. 2018 Jul 6;23(7):1649. doi: 10.3390/molecules23071649 (PMC6100033; doi:10.3390/molecules23071649)
Supplement: Supplementary file 1 [file molecules-23-01649-s001.pdf]

Supporting Information

Synthesis of Novel Fluorescent Ruthenium Complex Appending Ac_4GlcNAc Moiety by Click Reaction

Qi Cheng [1], Yalu Cui [2], Nao Xiao [1,*], Jishun Lu [2] and Chen-Jie Fang [1]

[1] Beijing Area Major Laboratory of Peptide and Small Molecular Drugs, Engineering Research Center of Endogenous Prophylactic of Ministry of Education of China, School of Pharmaceutical Sciences, Capital Medical University, Beijing 100069, China; xiaonao@ccmu.edu.cn (N.X.)

[2] Key Laboratory of Systems Biomedicine (Ministry of Education), and Collaborative Innovation Center of Systems Biomedicine, Shanghai Center for Systems Biomedicine (SCSB), Shanghai Jiao Tong University, 800 Dongchuan Road, Shanghai 200240, China; lujishun@sjtu.edu.cn (J.-S.L.)

* Correspondence: xiaonao@ccmu.edu.cn; Tel.: +86-10-8391-1524

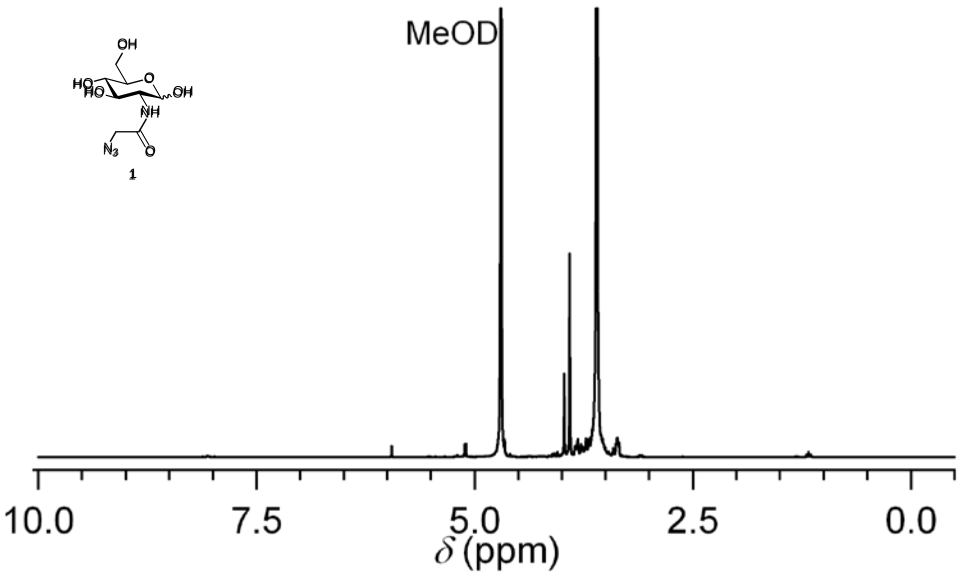

Figure S1. ^{1}H-NMR spectrum of **1** in MeOD.

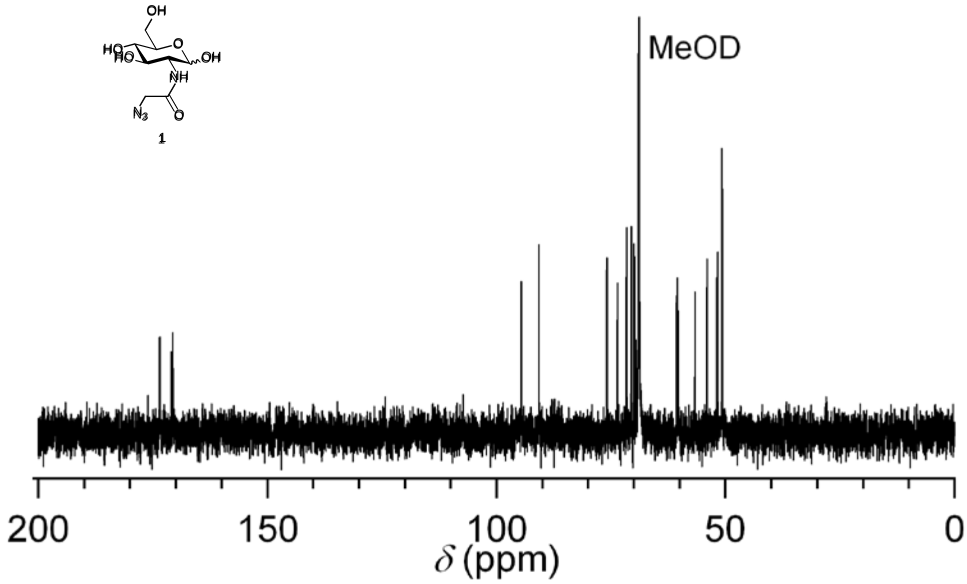

Figure S2. ^{13}C-NMR spectrum of **1** in MeOD.

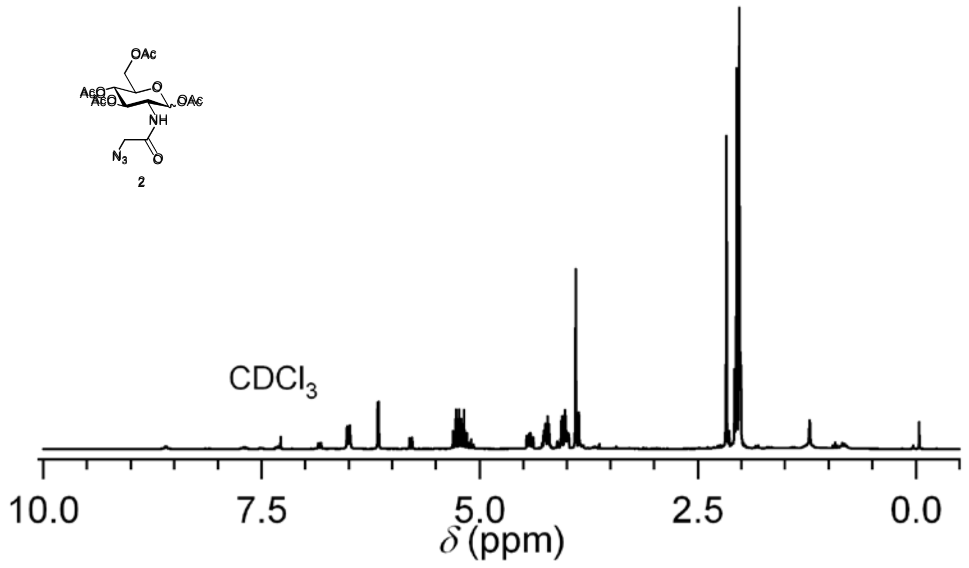

Figure S3. ^{1}H-NMR spectrum of **2** in $CDCl_3$.

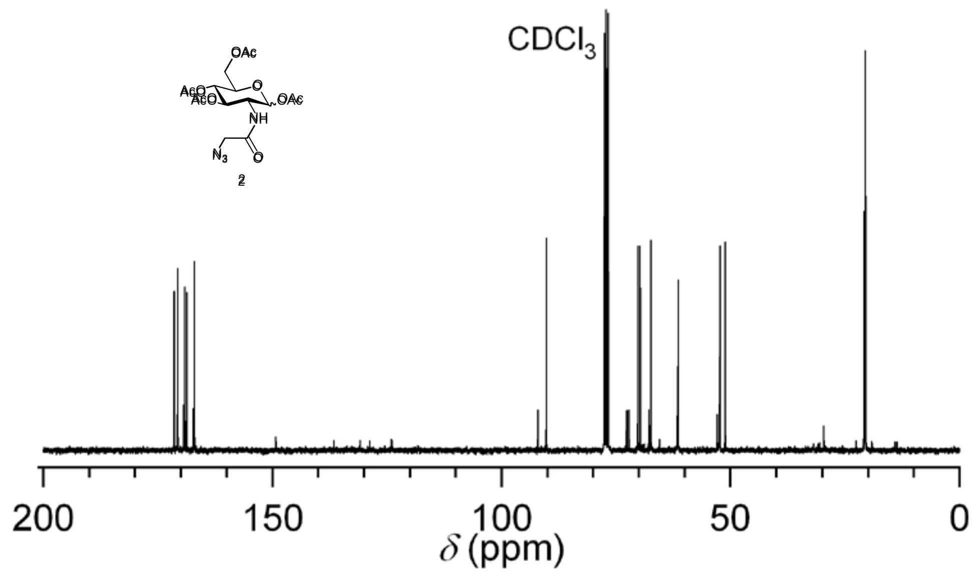

Figure S4. ^{13}C-NMR spectrum of **2** in $CDCl_3$.

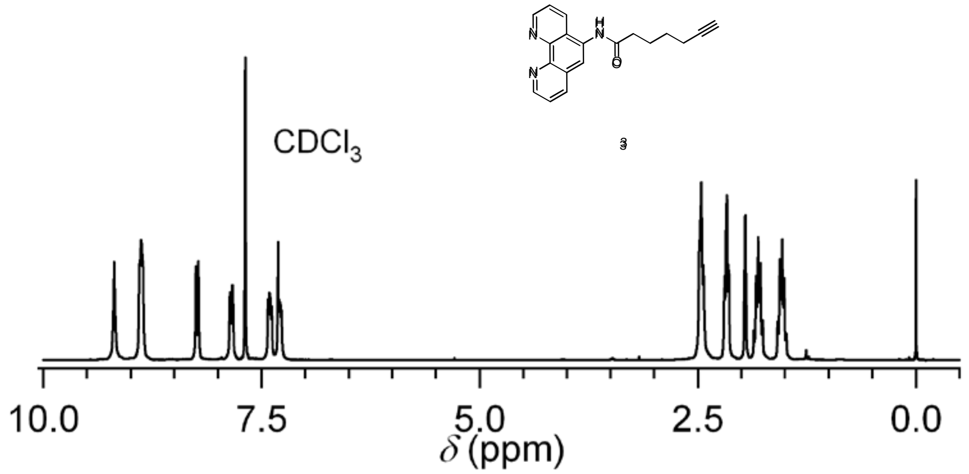

Figure S5. ^{1}H-NMR spectrum of **3** in $CDCl_3$.

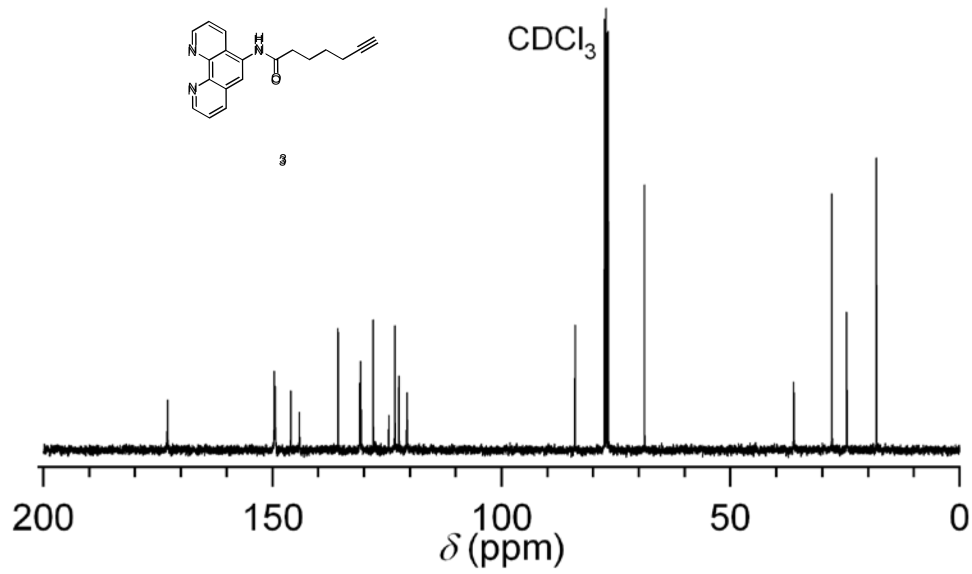

Figure S6. ^{13}C-NMR spectrum of **3** in $CDCl_3$.

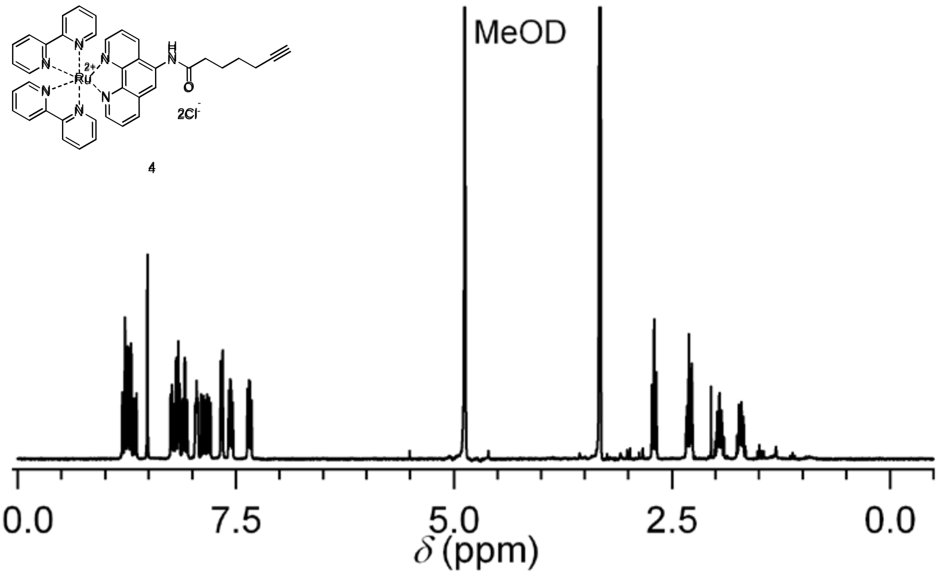

Figure S7. ^{1}H-NMR spectrum of **4** in MeOD.

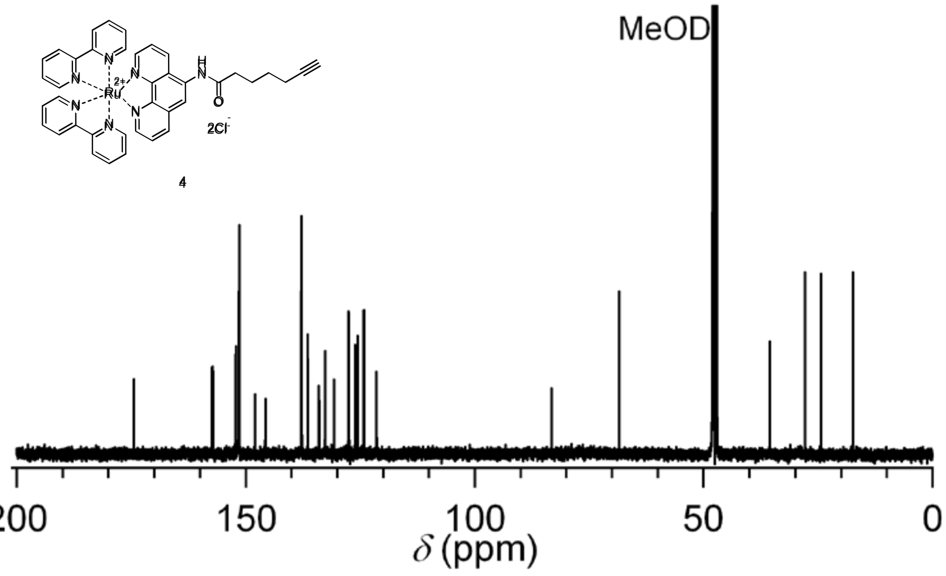

Figure S8. ^{13}C-NMR spectrum of **4** in MeOD.

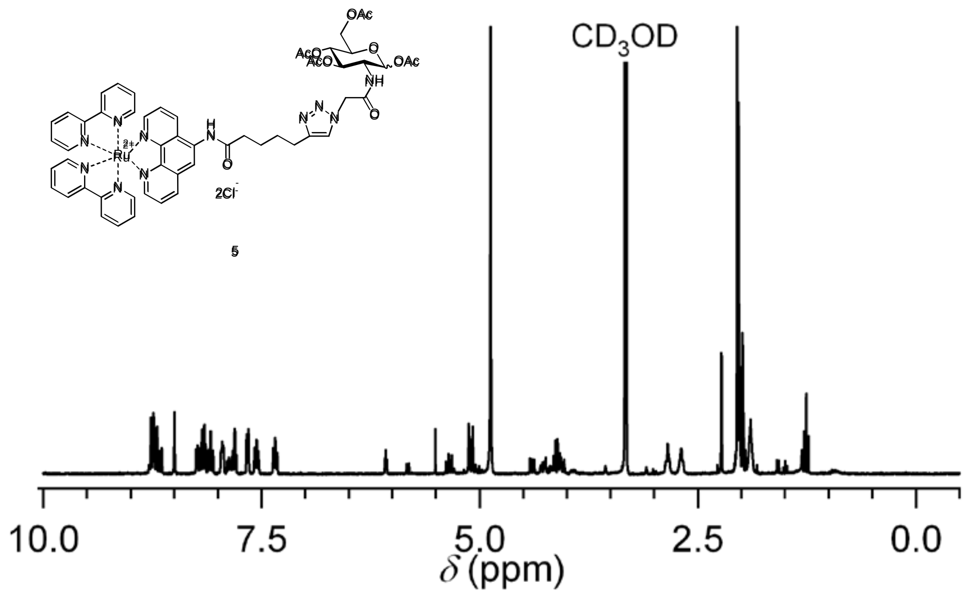

Figure S9. ^{1}H-NMR spectrum of **5** in MeOD.

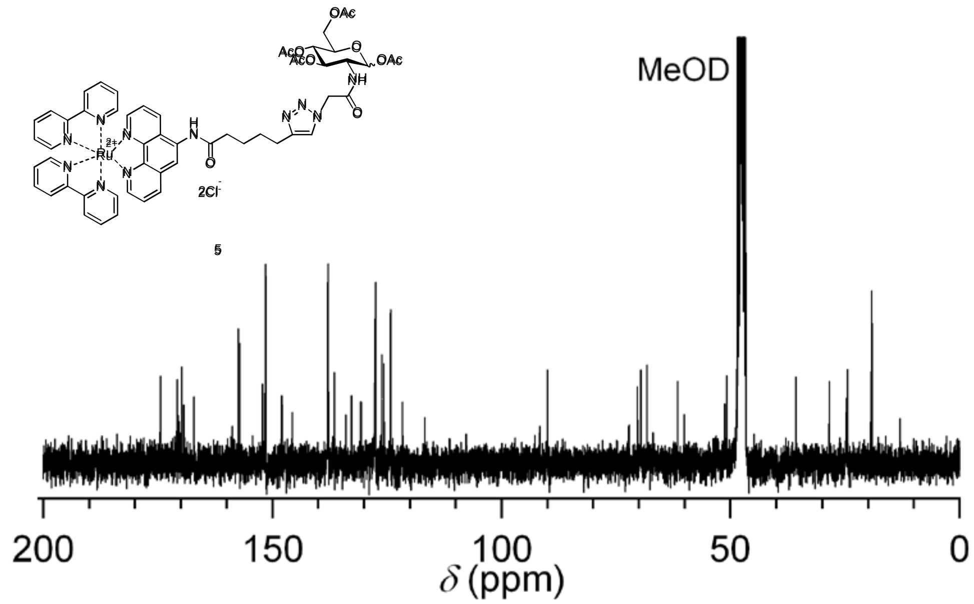

Figure S10. ^{13}C-NMR spectrum of **5** in MeOD.

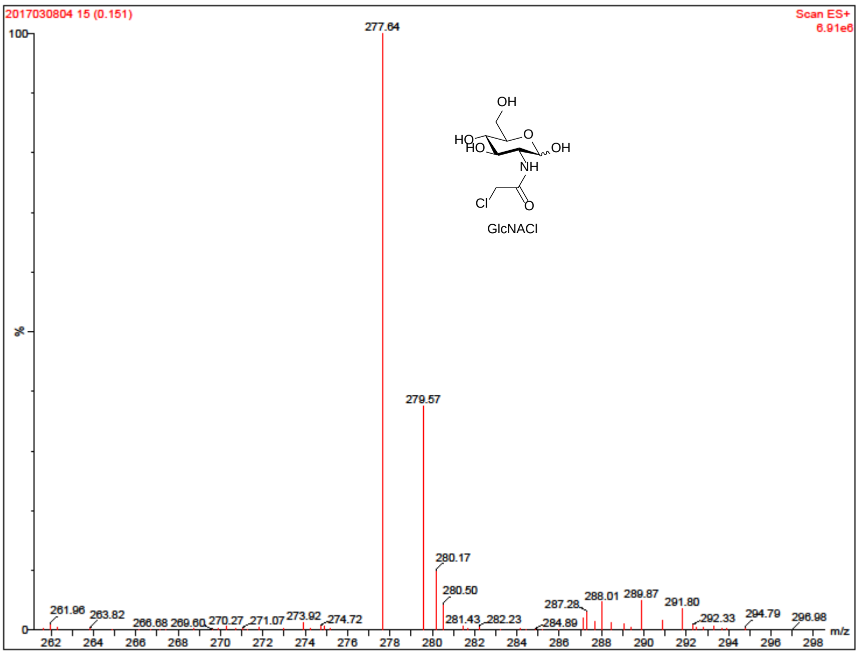

Figure S11. ESI-MS spectrum of GlcNACl in CH_3OH. ESI-MS (*m/z*): 277.64 $[M+Na]^+$ (Calcd. for $C_8H_{14}ClNNaO_6$: 278.04).

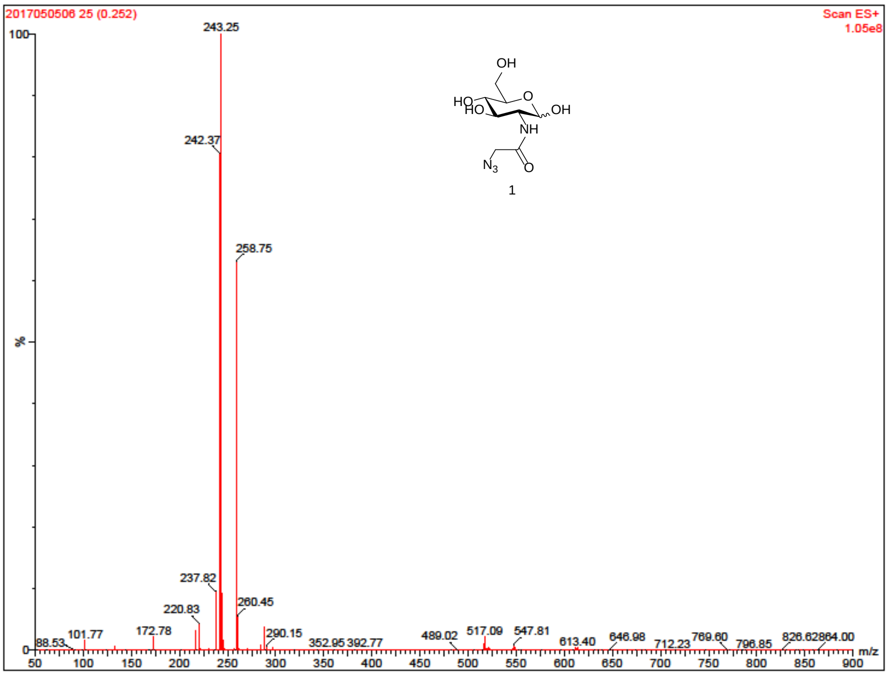

Figure S12. ESI-MS spectrum of **1** in CH_3OH. ESI-MS (*m/z*): 243.25 $[M-N_3]^+$ (Calcd. for $C_8H_{14}NNaO_6$: 243.07). ESI-MS (*m/z*): 258.75 $[M-N_2]^+$ (Calcd. for $C_8H_{14}N_2NaO_6$: 257.07).

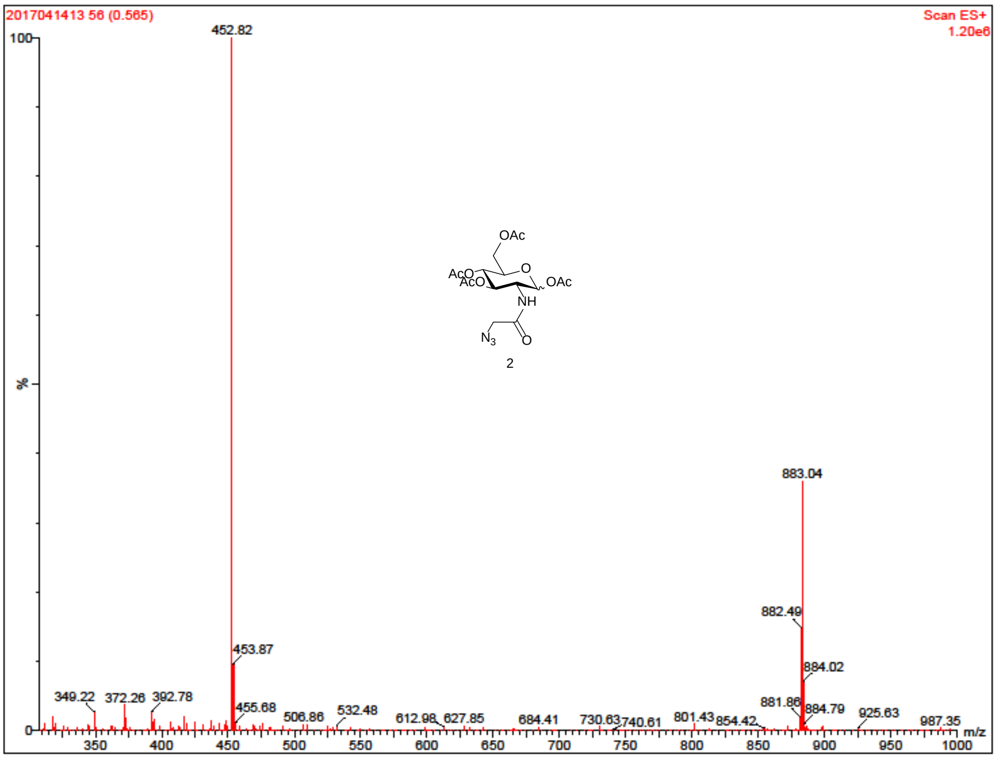

Figure S13. ESI-MS spectrum of **2** in CH_3OH. ESI-MS (*m*/*z*): 452.82 $[M+Na]^+$ (Calcd. for $C_{16}H_{22}N_4NaO_{10}$: 453.12). ESI-MS (*m*/*z*): 883.04 $[2M+Na]^+$ (Calcd. for $C_{32}H_{44}N_8NaO_{20}$: 883.26).

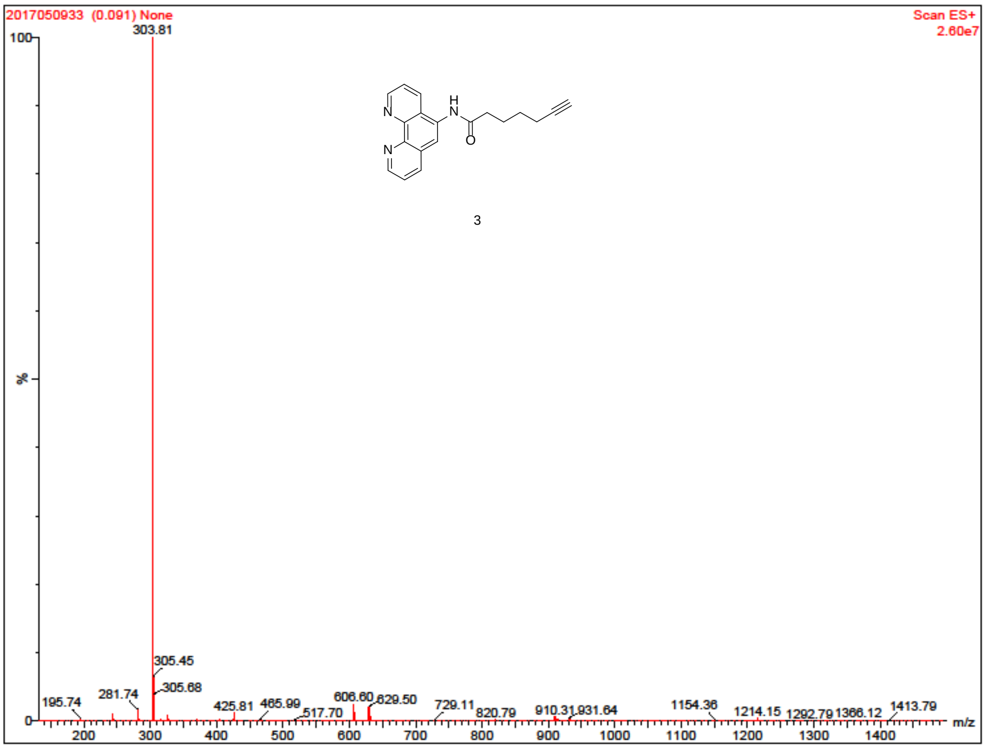

Figure S14. ESI-MS spectrum of **3** in CH_3OH. ESI-MS (m/z): 303.81 $[M+H]^+$ (Calcd. for $C_{19}H_{17}N_3O$: 304.14).

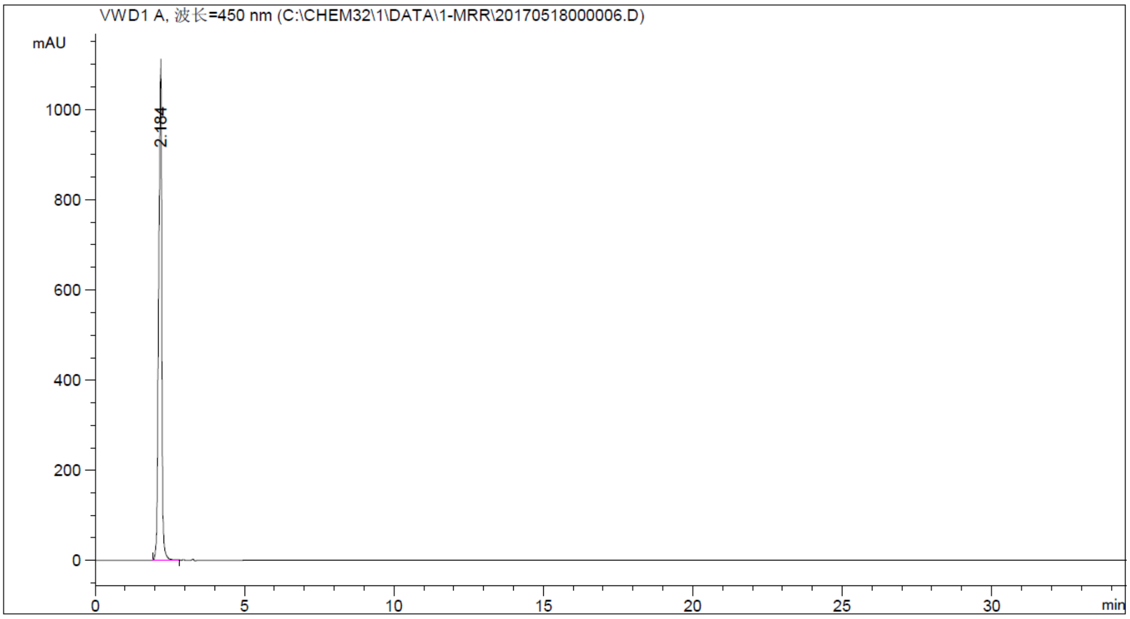

Figure S15. HPLC trace of **5** determined in $CH_3CN/H_2O/HAc$ (60:36:4). λ = 450 nm.

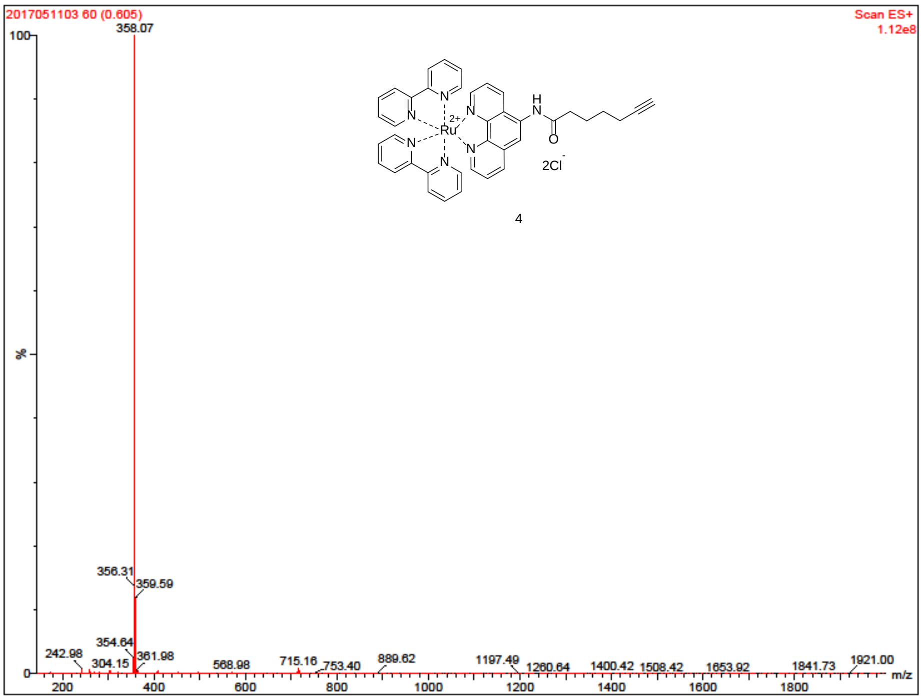

Figure S16. ESI-MS spectrum of **4** in CH_3OH. ESI-MS (m/z): $[M+H]^+$ (Calcd. for $C_{39}H_{34}Cl_2N_7ORu$: 788.12), 358.07 $[M-2Cl]^{2+}$ (Calcd. for $C_{39}H_{33}N_7ORu/2$: 358.59).

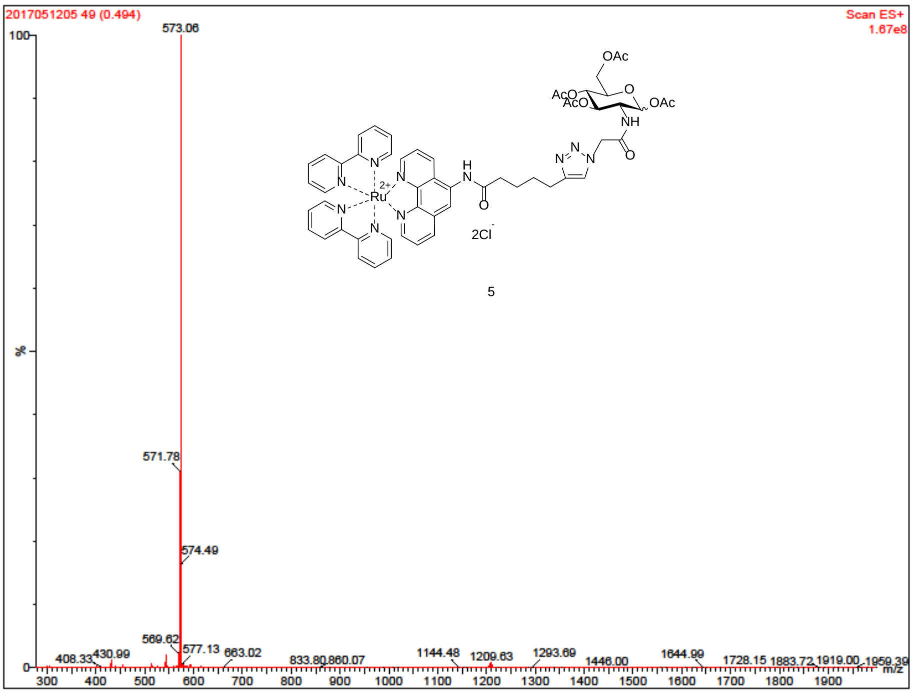

Figure S17. ESI-MS spectrum of **5** in CH_3OH. ESI-MS (m/z): $[M+H]^+$ (Calcd. for $C_{55}H_{56}Cl_2N_{11}O_{11}Ru$: 1218.26), 573.06 $[M-2Cl]^{2+}$ (Calcd. for $C_{55}H_{55}N_{11}O_{11}Ru/2$: 573.655)

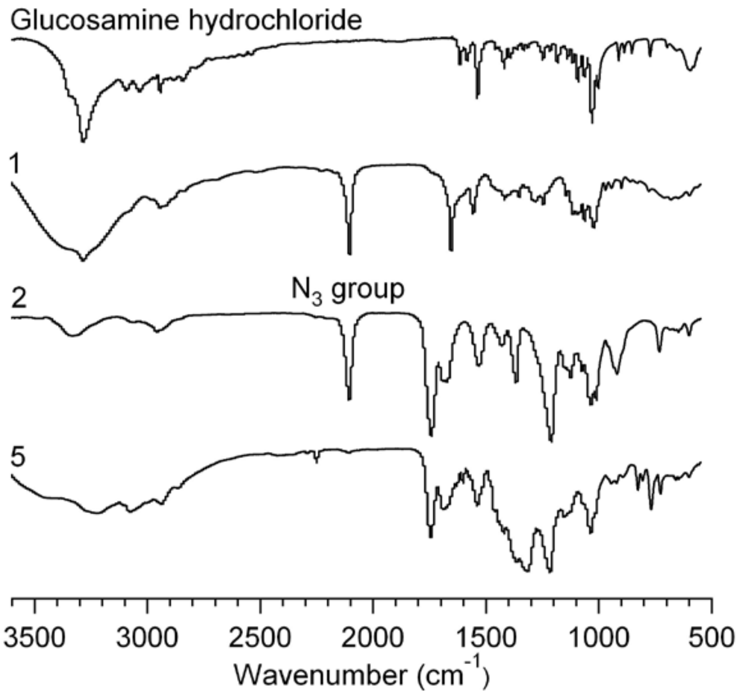

Figure S18. IR spectra of glucosamine hydrochloride, **1**, **2** and **5**.

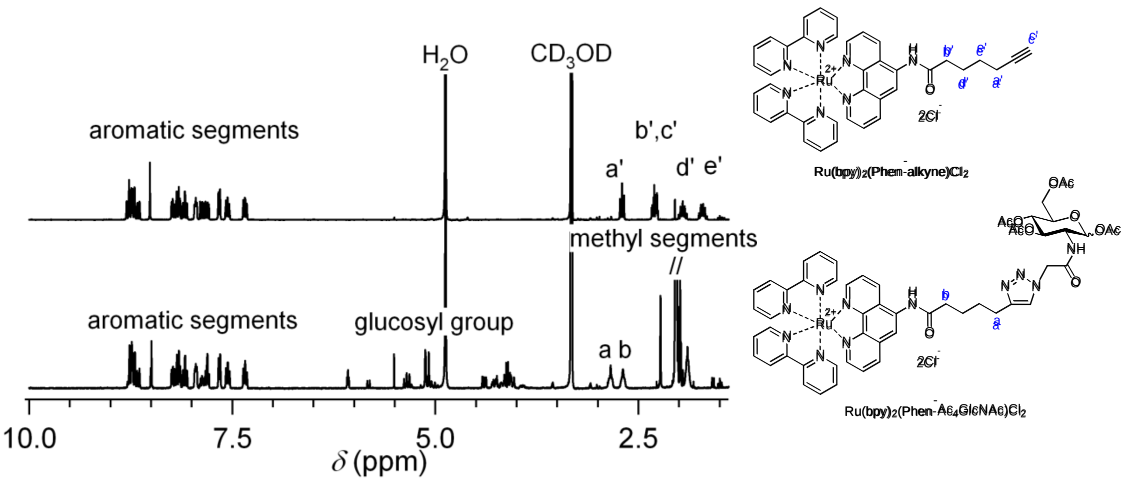

Figure S19. ^{1}H-NMR spectra of **4** and **5** in MeOD.

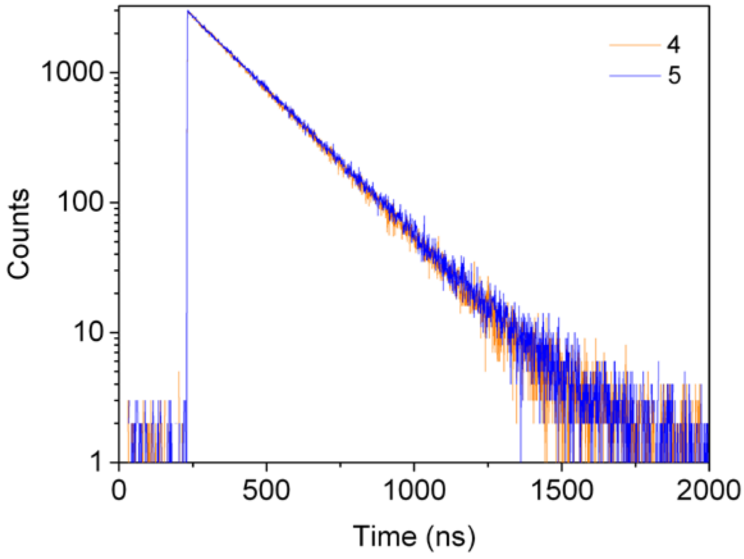

Figure S20. The fluorescent decay curves (collected at 590 nm) of **4** and **5** in MeOH solution.

Table S1. Photophysical properties of **4** and **5** were recorded in MeOH solution at room temperature. Decay times (τ) were presented in the table.

Ruthenium complexes	τ/ns	χ^2
4	190.4037	1.080
5	194.0749	0.993

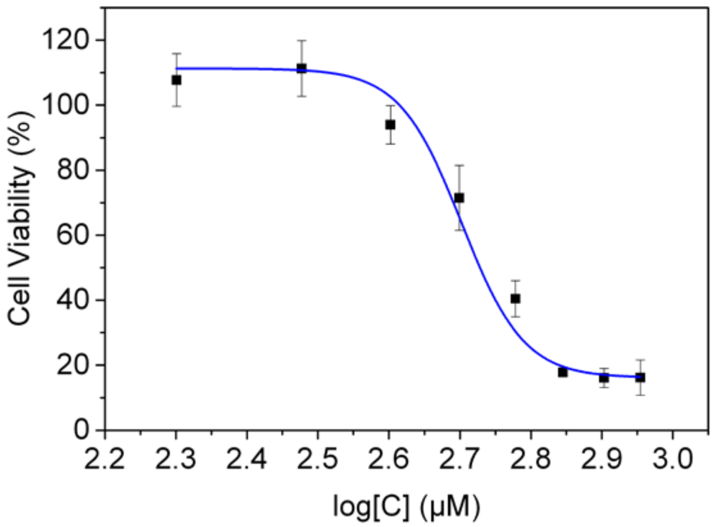

Figure S21. *In vitro* cell viability after incubation of MCF-7 cells with **5**.
